# Degradation of Acid Red 1 Catalyzed by Peroxidase Activity of Iron Oxide Nanoparticles and Detected by SERS

**DOI:** 10.3390/nano11113044

**Published:** 2021-11-12

**Authors:** Edna Vázquez-Vélez, Horacio Martínez, Fermín Castillo

**Affiliations:** Instituto de Ciencias Físicas, Universidad Nacional Autónoma de México. Av. Universidad 1000, Col. Chamilpa, 62210 Cuernvaca, Morelos, Mexico; ciro@icf.unam.mx

**Keywords:** magnetite, peroxidase activity, SERS

## Abstract

Magnetic iron oxide nanoparticles (MIONPs) were synthesized using tannic acid and characterized by Raman, FTIR, UV, and DRX spectroscopy. In a heterogeneous Fenton-like reaction, the catalytic peroxidase-like activity of MIONPs in the degradation of Acid Red 1 (AR 1) dye was investigated. TEM/STEM was used to determine the quasi-spherical morphology and particle size (3.2 nm) of the synthesized MIONPs. The XRD powder patterns were indexed according to the reverse spinel structure of magnetite, and SEM-EDS analysis confirmed their chemical composition. At pH = 3.5, the decomposition of H_2_O_2_ in hydroxyl radicals by MIONPs results in high AR 1 degradation (99%). This behavior was attributed to the size and surface properties of the MIONPs. Finally, the Surface Enhanced Raman Spectroscopy (SERS) technique detected intermediary compounds in the degradation process.

## 1. Introduction

The textile industry represents two-thirds of the use of dyes resulting in one of the main sources of contamination. Due to the high concentration of dye in the wastewater from these installations, it is not easy to treat it satisfactorily. It is estimated that 2% of the colorants are discharged directly into the aqueous effluent [1]. Azo-type dyes are the most commonly used polluting compounds, accounting for 70% of the total. Notably, the acid red 1 (AR 1) dye is among the 11 non-biodegradable azo compounds listed [2]. Nowadays, the importance of this type of dye increases in the field of biomedicine, electronics, and energy because dyes can absorb visible electromagnetic radiation with high efficiency. As a result, removing the non-biodegradable dye is a significant environmental issue. These compounds can be found in the aquatic environment, and they are poisonous to both marine and human organisms. Besides, they are mutagenic and carcinogenic [3]. For decades, there has been a growing interest in removing these difficult-to-remove compounds [4,5]. However, new technology is required to complete the mineralization of various dyes while minimizing their impact on the environment and humans.

Nanotechnology is one of the fastest-growing and emerging research areas today. Problems related to water quality can be solved or improved through it. The synthesis of nanoparticles is an economical, effective, efficient, and sustainable alternative. Its use makes treatment processes less polluting than traditional methods [6,7]. In recent decades, magnetic iron oxide nanoparticles (MIONPs) have sparked interest in catalytic wet peroxide oxidation (CWPO) or Fenton heterogeneous oxidation. The potential of these materials stems from their more remarkable ability to degrade recalcitrant contaminants, which involves the generation of hydroxyl radicals in sufficient quantities to allow oxidation [8,9]. The hydroxyl radical constitutes one of the most powerful oxidants (E0 = 2.73 V), and is much stronger than other conventional oxidizing species such as hydrogen peroxide (E0 = 1.31 V) or ozone (E0 = 1.52 V) [10]. MIONPs represent a promising alternative to the conventional catalysts used in CWPO due to their higher activity, easy recovery, and further reusability. In this context, one of the most critical challenges in CWPO is the development of more active and stable catalysts. Several studies on the degradation of recalcitrant dyes using MIONPs have been published [10]. Iron oxide nanoparticles have also been supported on various porous materials to improve their efficiency in heterogeneous Fenton-like catalysis [11]. Other studies have used ultrasonic radiation to improve the degradation efficiency of dyes [12]. The peroxidase-like activity of magnetite nanoparticles to generate hydroxyl radicals, in particular, has been extensively studied in the degradation of molecules [13]. However, its study on the degradation of recalcitrant contaminants continues to be of great interest due to being one of the most cost-effective CWPO.

This work describes an accessible synthesis of magnetic iron oxide nanoparticles (MIONPs) utilizing tannic acid, a water-soluble polyphenol frequently found in herbaceous and woody plants [14]. Furthermore, the peroxidase-like activity of synthesized MIONPs in the oxidative degradation of acid red 1 dye was investigated. The quantification of hydrogen peroxide generation confirmed that AR 1 was degraded by hydroxyl radicals. Finally, the SERS technique was used to characterize the intermediate compounds in the degradation process.

## 2. Materials and Methods

### 2.1. Materials

All chemical reagents and solvents were purchased from Merck KGaA, Darmstadt, Germany. Deionized water Millipore was used for NPs formation, and all degradation experiments were performed with ultrapure water. The acid red 1 solution was prepared fresh before each experiment to ensure the accuracy of the experimental data. The pH of the solution was adjusted using NaOH or HCl solution (5%).

### 2.2. Synthesis of MIONPs

Magnetic Iron oxide nanoparticles (MIONPs) were synthesized by dissolving 0.8 g (3 mmol) of iron (III) chloride hexahydrate (FeCl_3_·6H_2_O) in 20 mL of deionized water to which the capping agent was added: 0.25 g of tannic acid (0.14 mmol) dissolved in 20 mL of deionized water. The solution immediately produces a black-colored solution, which was mechanically stirred at 250 rpm. The pH of the solution was then adjusted to 7.6 ± 0.2 by adding NaOH solution drop by drop. The result was the formation of a magnetic black precipitate. The solid was washed three times with deionized water (40 mL) to remove the salts, once with acetone to remove residual water, and three times with ethanol to eliminate the organic matter. All washes were accomplished with the assistance of an external magnetic field to separate the liquid from the solid. Finally, the black solid was dried at 80 °C for two hours and used immediately for degradation tests.

### 2.3. Characterization Techniques

Morphological and size study of the nanoparticles was analyzed by field emission transmission electron microscopy (TEM/STEM, JEOL JEM-2010 FEG, Akishima, Tokyo, Japan) operating at 200 kV. A drop of the solution (NPs dispersed in EtOH) was placed on a carbon-coated copper grid to prepare the sample. Scanning electron microscopy (SEM) and energy-dispersive X-ray spectroscopy (EDS) was performed on MIONPs using a TESCAN MIRA 3 GMU microscope from Brno—Kohoutovice, Czech Republic; coupled with energy dispersive X-ray analysis (Brucker). A drop of the solution of NPs dispersed was placed on carbon tape to create the sample. The X-ray diffraction (XRD) patterns of the synthesized nanoparticles were recorded using a Rigaku Miniflex DMAX 2200 X-ray diffractometer, Austin, TX, USA. The solid was subjected to Cu Kα radiation (1.54 Å) with graphite monochromator in the 2θ range of 5–80°. For the characterization by UV-vis spectrophotometry, the solid was dispersed in ethanol with the help of ultrasonic vibrations. The solution was analyzed using an Ocean View UV-vis spectrophotometer, Orlando, FL, USA. FTIR spectroscopy was used to characterize the nanoparticles using a Bruker ATR-FTIR spectrometer Alpha II, Ettlingen, Germany. Raman spectroscopy using a confocal microscope Raman Bruker SENTERRA II from Ettlingen, Germany, confirmed the composition and crystallographic phase of Iron NPs. A low laser power (1 mW) was used to avoid sample degradation due to laser heating. The accumulation time was 10,000 ms. Bleaching of 1 mW and 100 ms was performed to attenuate the fluorescence of the sample. A power of 25 mW and an integration time of 20,000 ms were used for the samples analyzed by the SERS technique.

### 2.4. Degradation Procedure

The pH effect on dye degradation was studied by adjusting the starting pH of the AR 1 solution at a concentration of 50 mg/L. AR 1 solution was carried to a pH = 3.5, 4.5, 6.5, 8.5, and 10.5 using either solution of HCl or NaOH (5%). MIONPs (2 g/L) were dispersed in AR 1 solution (50 mL) using ultrasonic vibrations for 5 s. A 2 mM concentration of H_2_O_2_ was then added to begin heterogeneous Fenton-like catalysis. This amount was found to be the bare minimum required to initiate the peroxidase-like activity of MIONPs. The oxidation process was carried out in the absence of light. The absorbance was measured at 505.06 nm after 60 min of treatment. On the other hand, the degradation of AR 1 was investigated according to the treatment time. For this, 50 mL of a 200 mg/L solution was prepared. This concentration required a minimum of 4 g/L of MIONPs to start the catalytic oxidation. At the end of the process, MNIONPs were removed from the solution using a magnet. After the first degradation cycle, the solid was dried at 90 °C and immediately reused for the second cycle of degradation under the same conditions.

### 2.5. UV-Vis Spectroscopic Analysis

The concentration of AR 1 was measured at 505.06 nm by UV-vis spectrophotometry, using an Ocean View UV-Vis spectrophotometer. Besides, the concentration of H_2_O_2_ during the degradation process was quantified using titanium sulfate spectrophotometry [15]. The absorbance of this solution was measured at 400.13 nm. Previously, a calibration plot based on Beer–law Lambert’s equation was established by relating the absorption to AR 1 and the hydrogen peroxide concentration. AR 1 dye degradation efficiency (%) was calculated using equation 1, where Ci is the initial concentration, and Cf is the final concentration of solution after degradation:

(1) $$\mathrm{Degradation}\;\left(\%\right)=\frac{\left(\mathrm{Ci}-\mathrm{Cf}\right)}{\mathrm{Ci}}.\;100$$

## 3. Results and Discussion

### 3.1. Characterization of MIONPs

**Figure 1:**
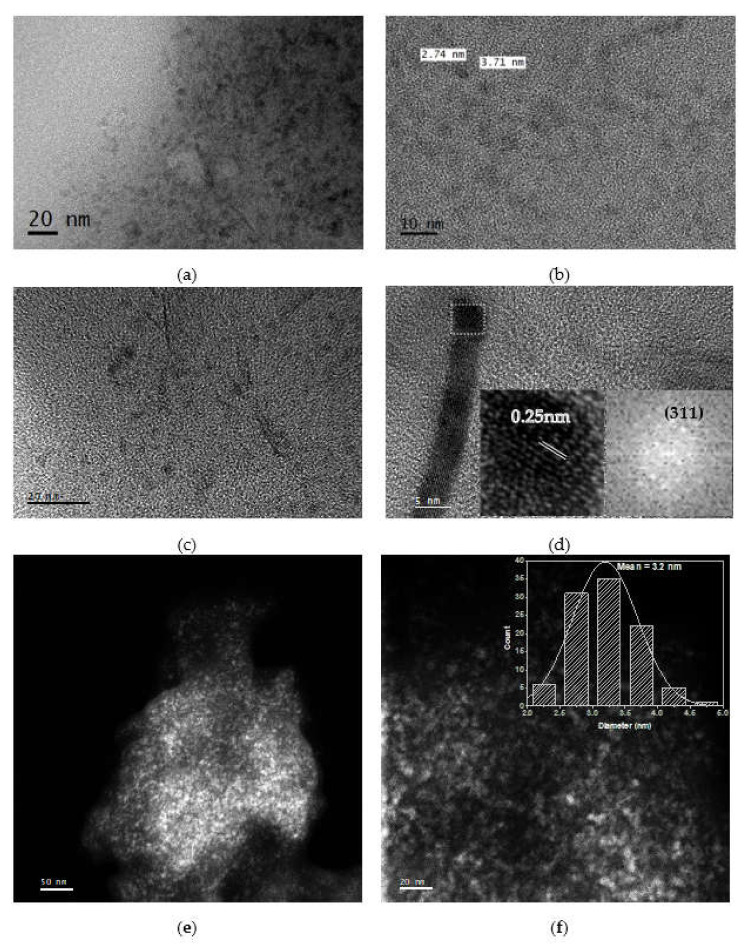
(**a**–**d**) TEM images and (**e**–**f**) STEM images of synthesized MIONPs.

The morphology and size of the synthesized MIONPs were analyzed by TEM/STEM images (Figure 1). Figure 1a,b shows TEM images of nanoparticles with a quasi-spherical morphology and a size of about 3 nm. MIONPs can aggregate and form large particles due to their strong anisotropy dipolar [16], so certain needle forms are observed in Figure 1c. Using the Digital Micrograph (DM) software 3.7 Gatan, Pleasanton, CA, USA, one large aggregate (Figure 1d) was analyzed and digitally processed to obtain fast Fourier transform (FFT). The bright circular rings indicate the polycrystalline phase in the selected area electron diffraction (SAED) patterns. The spaced-resolved lattice fringes with an interplanar distance of 0.25 nm agree well with the lattice spacing of Fe_3_O_4_ (311) planes [11]. Figure 1e,f shows the STEM analysis of NPs; these images revealed a better quasi-spherical morphology. The size of NPs in Figure 1d was analyzed to perform a histogram employing the DM software, which confirms an average size of 3.2 nm.

**Figure 2:**
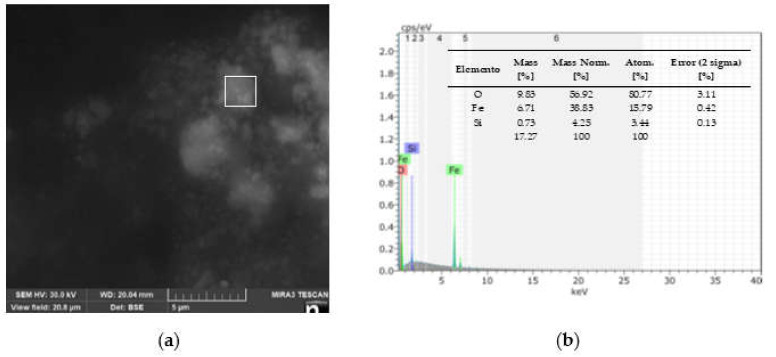
(**a**) SEM image and (**b**) EDS spectrum of MIONPs.

The SEM image is seen in Figure 2a with its point EDS analysis applied in the box marked. The EDS spectrum recorded for the nanoparticles is shown in Figure 2b, where a strong signal for elemental iron is observed at 6.2 keV and another small signal at 7.1 keV. The presence of an oxygen peak at 0.6 keV indicates that iron oxides were formed. The mass relationship corresponds to Fe_3_O_4_. Similar results were obtained using Ridge gourd peel extract [17] to synthesize magnetite nanoparticles.

**Figure 3:**
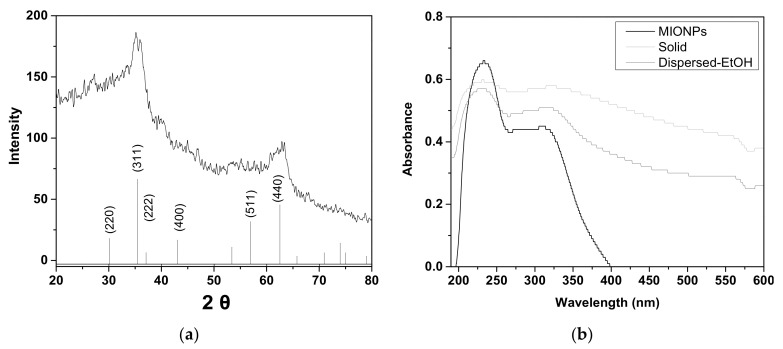
Spectra of MIONPs: (**a**) XRD and (**b**) UV-visible.

Figure 3a shows the XRD spectrum of synthesized MIONPs. The broad diffraction peaks confirm the formation of an amorphous solid. Here, the width in the middle of the maximum is related to the smallest particle size [18]. The diffractogram depicts the initial crystallizing phase, which corresponds primarily to the magnetite phase [19]. The diffraction patterns are according to the inverse spinel structure of magnetite shown in down of figure. The diffraction pattern card from magnetite powder (19–0629) was obtained from Columbian Carbon Co., in New York, USA. The five characteristic peaks at 2 = 30.50, 35.87, 43.65, 57.54, and 63.28° are assigned to (220), (311), (400), (511), and (440) crystalline planes, respectively. However, it is well established that maghemite (γ-Fe_2_O_3)_ and magnetite (Fe_3_O_4_) reveal similar XRD profiles [20]. Bibi et al. [21] published a diffractogram of γ-Fe_2_O_3_ Nps as well as its absorbance spectrum, which showed a peak at 371.71 nm. Compared to this study, MIONPs revealed a different diffractogram in their intensities. Figure 2b depicts the absorbance spectrum showing a peak at 228 nm, consistent with previous reports for the magnetite phase [22].

UV-visible spectroscopy was performed to analyze the stability of magnetite nanoparticles. Figure 3b depicts the absorbance spectrum of MIONPs in a solid-state after two days of conservation in a closed container without an inert atmosphere. The peak at 228 nm decreases, while a broad peak of 350–600 increases, indicating a change to the magnetite phase. However, the same figure also shows the spectrum of MIONPs conserved in an anhydrous ethanol dispersion after fifteen days. The peak at 228 nm is still visible, but the broad peak at 350–600 nm appears. The peak at about 350 nm is assigned to octahedral Fe^3+^ in small oligomeric FeO_x_ clusters, and the bands at 450−600 nm are characteristic of the Fe_2_O_3_ aggregates [23]. These results indicate that MIONPs can be stable in the dispersion of anhydrous ethanol during a particular time.

**Figure 4:**
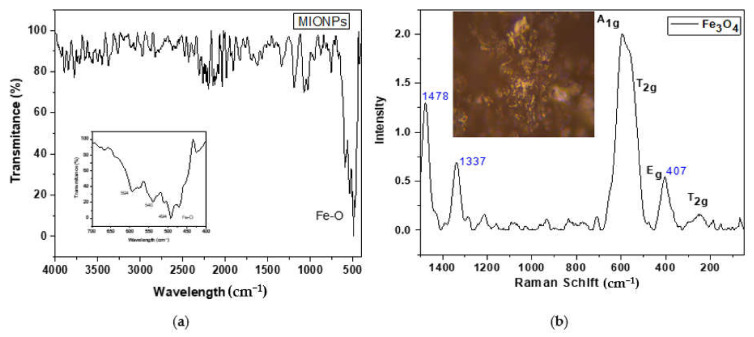
(**a**) FTIR and (**b**) Raman spectra with optical imagen at 50× magnification, of MIONPs.

ATR-FTIR analysis was performed to observe the surface purity of the MIONPs, see Figure 4a. According to the literature, the magnetite FTIR spectrum has two strong absorption bands at 570 and 390 cm^−1^, which can be attributed to the Fe-O stretching mode of the tetrahedral and octahedral sites, respectively [24]. In this study, the Fe-O vibration signal of MIONPs was observed at 540 and 494 cm^−1^. This shift can be attributed to the small size of the nanoparticles [25]. However, a weak shoulder was observed at 594 cm^−1^, which could be due to the beginning of the γ-Fe_2_O_3_ formation, with absorption bands at 630, 590, and 430 cm^−1^ [24]. Nevertheless, none of the vibration band was observed around 3400 cm^−1^, indicating the vibration stretching of the O-H group from tannic acid. So, the MIONPs used for the degradation test was free of organic matter.

On the other hand, in Raman spectroscopy, magnetite has a spinel structure, and five phonon bands have been theoretically predicted: one from A_1g_, a second from E_g_, and three from T_2g_ [26]. Zhang et al. reported the Fe_3_O_4_ nanoparticles Raman spectrum. The T_2g_ modes were observed at 305 and another at 534 cm^−1^, the E_g_ mode at 513 cm^−1^, and the A_1g_ mode at 660 cm^−1^ [20]. The Raman spectrum of synthesized MIONPs is shown in Figure 3b. The band assigned to the A_1g_ mode was shifted to 602 cm^−1^ due to particle size. However, Profile Breit Wigner Fano (BWF) [18] can better describe this shift to low frequency. Nevertheless, the phonon mode (A_1g_) of γ-Fe_2_O_3_ was not observed at 710 cm^−1^, implying no phases coexist on the surface of MIONPs [19]. On the other hand, the peaks at 407, 1337, and 1478 cm^−1^ indicate the start of the oxidation to hematite by laser action. This fact is due to the increased temperature of laser-heated spots results in a softening of phonon frequency [27].

MIONPs were successfully synthesized by the coprecipitation method using tannic acid at pH < 8. Synthesis of magnetite nanoparticles at pH = 8 has been reported using a capping agent synthesized from gallic acid [28]. However, the formation γ-Fe_2_O_3_ from the synthesis with tannic acid has also been described at pH = 12 [29]. Then, the pH control in the magnetite synthesis plays an essential role in controlling the oxidation of Fe^2+^ to Fe^3+^ [30].

### 3.2. Degradation Catalyzed by MIONPs

**Figure 5:**
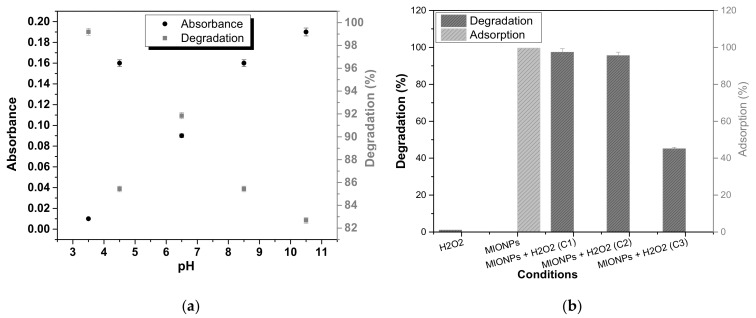
Graphic of (**a**) absorbance and degradation (%) at different pH and (**b**) degradation and adsorption at different conditions of the reaction.

Figure 5a shows the absorbance value of the AR 1 solutions treated at different pH and their corresponding percent degradation in heterogeneous Fenton-like catalysis. The most representative value was the absorbance of 0.01 for the solution at pH = 3.5 with 99.12% of degradation efficiency. These findings are consistent with the Fenton reaction, which shows that the highest degradation efficiency occurs at pH = ~4. The degradation efficiency at pH > 4 is around 85%, except at pH = 6.5, which is 92%. The oxidation process produces inactive ferric oxyhydroxides at pH > 4, which reduces degradation efficiency. However, a different behavior was observed when the pH = 6.5. Here, the inherent pH of the solution prevented the beginning formation of oxyhydroxides. Furthermore, the adsorption of the dye on Fe^3+^ probably occurs through a ligand with the non-protonated carbonyl groups of AR 1. The geometry of the molecule adsorbed on MIONPs surface through a complex plays an essential role in the activation of reactive species, like it has been reported for EDTA complex, which generates hydroxyl and oxygen radicals [31].

**Figure 6:**
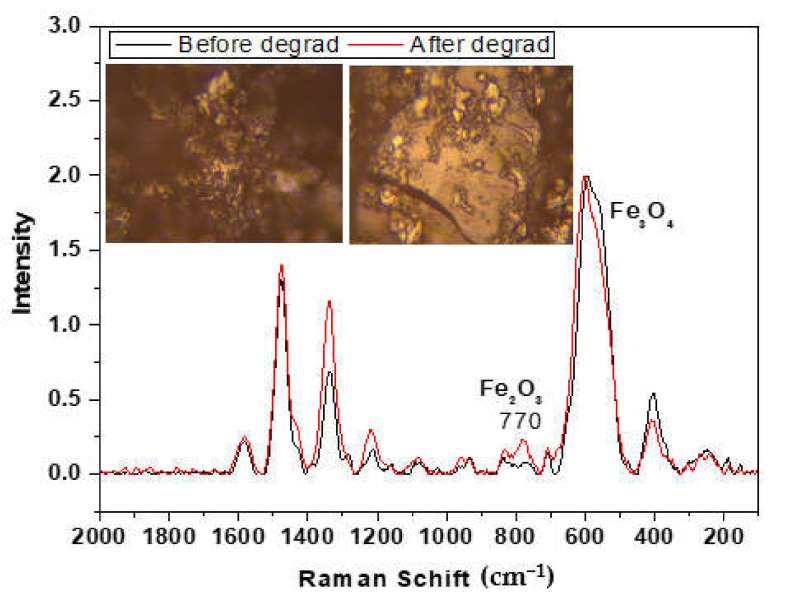
Raman spectra of MIONPs before and after AR 1 degradation.

Figure 5b shows the degradation percentage of AR 1 in an aqueous solution at 200 mg/L under different conditions after 60 min of treatment. The adsorption of AR 1 by MIONPs was 99.5% after 10 min of their addition. The addition of only H_2_O_2_ did not achieve the AR 1 degradation. The dilution of the solution by adding H_2_O_2_ caused just an instantaneous decrease in the AR 1 concentration. However, the oxidation process immediately occurred when H_2_O_2_ was introduced into the system to initiate the catalytic reaction following AR 1 adsorption on MIONPs. The degradation of AR 1 reached 97.4%. MIONPs stability and reusability were studied because of the essential use for their application. In the second degradation cycle (C2), the efficiency decreased to 96% and 45% in the third cycle (C3). Figure 6 shows the Raman spectrum of MIONPs before and after of AR 1 degradation process. The spectrum after degradation corresponds mainly to the magnetite phase, but the maghemite phase begins to be observed at 770 cm^−1^. In the optical images of MIONPs, a change in porosity, shape, and aggregation state was detected after the treatment process, which corroborates that the MIONPs act as adsorbents and exhibit catalytic activity for decomposition H_2_O_2_.

### 3.3. Peroxidase-like Activity of MIONPs

**Figure 7:**
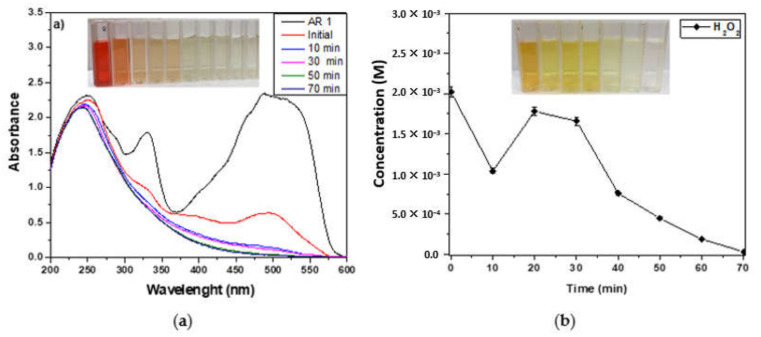
(**a**) UV-Visible spectra of AR 1 and (**b**) concentration of H_2_O_2_ in the degradation process.

The degradation of AR 1 as a function of time is shown in Figure 7a for a concentration of 200 mg/L. This concentration is mainly reported for effluents that need to be treated [1]. The double peak with a maximum at λ = 531 and 505.5 nm is the characteristic band of the conjugated electron structure (chromophore group) corresponding to the π-π* transition bond of N=N, while the second peak at about 325 nm corresponding to the π-π* bond of N-H. The 240 nm and 280 nm are the bands due to benzene and naphthalene rings [32]. The solution’s absorbance was measured at 505 nm. Figure 7a depicts the discoloration image of solutions treated each 10 min, with their absorbance spectra. The graph shows that the double absorption peak at 505 nm and 531 nm decreases with increasing time, indicating that AR 1 degradation is effective. The intensity of this band significantly reduced after the addition of H_2_O_2_ (initial). The highest percentage of degradation (84%) occurs within the first 10 min, and degradation was 97.4% completed up to 40 min. UV-visible spectra indicate the AR 1 degradation to small molecules from the chromophore groups.

Figure 7b shows the concentration of H_2_O_2_ quantified during the heterogeneous Fenton-like catalysis depicted in Figure 7a. The titanium sulfate spectrophotometry method was used to determine the concentration of peroxide. It was observed that the H_2_O_2_ decreased in the first ten minutes, which corresponded to the highest degradation seen in the first minutes after initiation with hydrogen peroxide. After, H_2_O_2_ generation catalyzed by MIONPs is followed in the next 10 min to achieve the maximum dye degradation. Finally, the peroxide consumption is observed slowly and concludes in 50 min more.

### 3.4. Mechanism of AR 1 Degradation

Peroxidases are a group of enzymes capable of catalyzing the oxidation of hydrogen peroxide into hydroxyl radicals. These radicals further participate in electron exchange with substrates producing color on oxidation. This activity is performed by the HEMO group (a Fe ion coordinated with a porphyrin that acts as a tetradentate ligand), which is found in peroxidase-type enzymes. Gao et al. [33] reported on the peroxidase-like activity of magnetite nanoparticles in the presence of peroxide. However, it has been stated that Fe_3_O_4_ MNPs are not as effective in the treatment of hazardous organic pollutants. It is necessary to increase the H_2_O_2_ activation capacity of Fe_3_O_4_ MNPs, with the assistance of ultrasonic irradiation [13]. In this study, we investigate the peroxidase-like activity of MIONPs in the oxidation of a recalcitrant dye in the absence of assisted irradiation. The reaction mechanism of MIONPs in the generation of OH radicals is due to their actuation as redox enzymes by the electron exchange escalated by their superficial atoms. Besides, when the organic molecule (AR 1) is adsorbed on the surface of MIONPs through electronic interaction at the molecule-metal interface to form a monolayer, these electrostatic and steric interactions promote the catalytic activity on its degradation. Then, the redox reaction occurs on the surface of MIONPs, but is related to the heterogeneous Fenton reaction in solution through the following equations [31]:

Fe^2+^ + H_2_O_2_   ➞   Fe^3+^ + ^●^OH + OH^−^ (2)

Fe^3+^ + H_2_O_2_   ➞   Fe^2+^ + ^●^OOH + H^+^ (3)

HO_2_^●^ + HO_2_^●^   ➞   H_2_O_2_ + O_2_ (4)

^●^OH + AR 1   ⬌   intermediates   ⬌   CO_2_ + H_2_O (5)

AR 1 oxidation begins when the bare minimum of H_2_O_2_ is added to the dye solution to begin the catalytic activity of MIONPs. This relationship is not stoichiometric, unlike the conventional Fenton reaction. Then, the degradation of AR 1 occurs mainly at the solid-liquid interfaces of MIONPs, where the formation of hydroxyl radicals is due to the catalyzed decomposition of hydrogen peroxide by the active sites (Fe^2+^ and Fe^3+^) of MIONPs.

**Figure 8:**
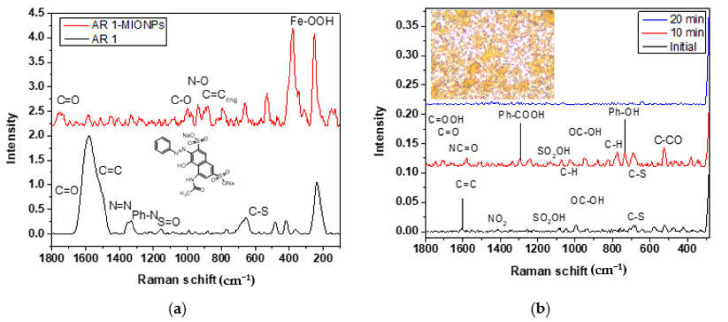
Raman spectra of (**a**) AR 1 (down) and AR 1 adsorbed on MIONPs (up); (**b**) intermediaries’ compounds, and optical image at 50x magnification of the SERS substrate (initial).

Figure 8a shows the Raman spectrum of AR 1 (down), where a broad and strong peak was observed at 1580 cm^−1^ corresponding to the vibrational signal C=C of the aromatic rings. The carbonyl signal of the amide group coexists in this peak because the electric field from the aromatic ring causes a shift at a low frequency. The azo group N=N signal was detected at 1353 cm^−1^, and the aromatic ring C-N bond vibration signal was detected at 1330 cm^−1^. The sulfate group S=O and the vibration signal from the aromatic ring C-S bond were observed at 1156 cm^−1^ and 656 cm^−1^. The AR 1 spectrum (up) adsorbed on the surface of MIONPs showed the signal of C=O from the amide group displaced at 1748 cm^−1^ because of electrostatic interaction with MIONPs. The vibrational signal from the aromatic ring occurs at C=C at 1580 cm^−1^, but another peak corresponding to this signal appears at 880 cm^−1^. This unusual signal is caused by a change in the symmetry of the molecule that interacts with the MIONPs surface. Finally, two vibrational signals appear that correspond to the C-O and N-O bonds, most likely as a result of AR 1 forming a coordination ligand with Fe^2+^ through the amide and azo groups. The strong signals at 253 and 375 cm^−1^ correspond to γ-FeOOH [26].

### 3.5. Compounds Detected by SERS in AR 1 Degradation

SERS analysis was performed on solutions obtained from Figure 7a. To detect AR 1, the initial and treated solutions were deposited on Ag nanoflowers [34] (on Si wafer substrate). Figure 8b shows the spectra of the initial, at 10 min, and 20 min solutions from the degradation process. After this time, no signal was detected for the following solutions. The spectra present the signals corresponding to different functional groups linked to intermediaries’ compounds reported and detected by mass spectroscopy during the H_2_O_2_-photolysis degradation of AR 1 [32].

**Figure 9:**
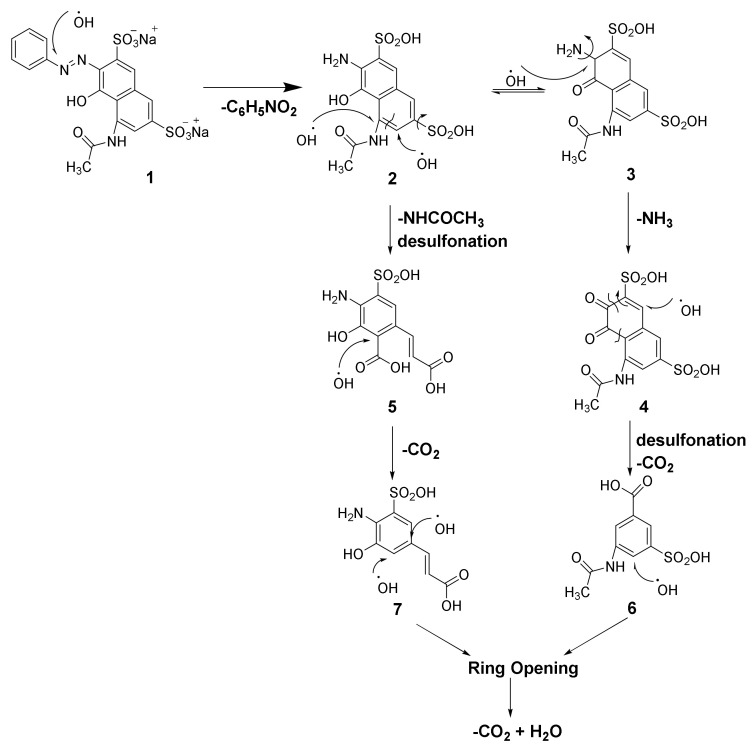
AR 1 degradation mechanism through oxidation by radicals ^●^OH.

Figure 9 shows the AR 1 degradation mechanism through oxidation by radicals ^●^OH. In the initial spectrum (immediate analysis after adding peroxide), the signal from azo group N=N at 1353 cm^−1^ in compound 1 was not observed. Instead, the vibrational signal for the NO_2_ group was detected at 1412 cm^−1^ because of N=N bond cleavage to obtain compound 2 and nitrophenol (C_6_H_5_NO_2_). Besides, the signal of the SO_2_OH group in intermediaries’ compounds was detected at 1084 cm^−1^. The spectrum corresponding to ten minutes of treatment revealed more signals with higher intensities from the functional groups in compounds 4, 5, 6, and 7. The signal corresponding to the C=O from carboxylic acid in compounds 5, 6, and 7 was detected at 1747 cm^−1^; the vibrational stretching of the C=O bond in compound 4 was observed at 1706 cm^−1^, and the signal of C=O from the amide group in compounds 3, 4, and 6 was seen at 1660 cm^−1^. At 1580 cm^−1,^ the vibrational signal was observed at C=C from the aromatic rings. At 1291 cm^−1^ and 731 cm^−1^, the benzoic acid and phenol peaks were detected, respectively. Then, the main intermediaries’ compounds in the degradation of AR 1 can be observed after ten minutes of catalytic activity of MIONPs. However, signals in the spectrum of the solution after 20 min of treatment decrease considerably.

The results obtained demonstrate that the application of MIONPs in AR 1 degradation suggests the superiority of the conventional Fenton-like catalysts through a dye highly recalcitrant. In this context, several studies on the degradation of azo-type organic dyes using iron oxide nanoparticles in heterogeneous Fenton-like catalysis reactions have been published. Table 1 depicts a comparison of those studies and this work. The peroxidase-like activity of MIONPs was reported for the degradation of Rhodamine (RhB) using ultrasonic irradiation (UI) [13,35]. The efficiency of degradation (90%) is due to the synergy of the ultrasonic and Fenton processes. However, this technology has the disadvantage of ultrasonic systems of high power consumption. Alternatively, magnetite nanoparticles supported on graphene oxide (GO) presented a good efficiency in degrading Acid orange 7 (AO 7) in normal conditions without irradiation. The Magnetite NPs without support of GO showed the same efficiency after 45 min of the process achieving 80%, and those supported in GO reached 98% of AO 7 degradation after three hours [11]. Nanocomposites of stable hematite nanoparticles with SiO_2_ have also been reported for heterogeneous Fenton-like catalysis. The degradation efficiency was excellent, but the treatment times were too long [23,36]. Compared to the other reported results, the synthesized MIONPs in this work present an excellent peroxidase-like activity in degrading a recalcitrant contaminant at a high concentration. The high efficiency of degradation under normal temperature conditions without irradiation is one of the study’s advantages. Besides, the particle size (3.2 nm) of MIONPs and the adsorption of AR 1 via—interactions from the aromatic ring structure on its basal plane and NPs surface contribute to the efficiency of this work. This dye adsorption increases the local concentration of AR 1 within the vicinity of the active sites to be further oxidized by the generated hydroxyl radicals.

## 4. Conclusions

Synthesis MIONPs with tannic acid is a good and efficient method in the obtention of magnetite nanoparticles. The nanoparticles were characterized and found to be 3.2 nm in average size, with a quasi-spherical morphology. The various spectroscopies and microscopies showed their chemical composition and magnetite phase. The peroxidase-like activity of the synthesized nanoparticles was verified by a heterogeneous Fenton-like catalysis reaction involving the degradation of AR 1. A high concentration of AR 1 was mineralized with an efficiency of 97.4% in one hour. MIONPs activity is strongly dependent on their size, shape, and surface structure; these characteristics can be appropriately tuned during their synthesis. Furthermore, the SERS technique identified the intermediary compounds in the degradation process. In the future, these nanoparticles could be supported in other nanomaterials to investigate their efficiency in heterogeneous Fenton-like catalysis, which would address the disadvantage of NPs instability and aggregation.

## Figures and Tables

**Table 1:** Degradation studies of azo dyes using different nanomaterials and process conditions.

Organic Dyes [Concentration]	Catalyst	Conditions	Degradation And Time	Reference
RhB (10 mg/L)	Fe_3_O_4_ NPs (10–15 nm)	Cat = 0.5 g/L, H_2_O_2_ 40 mM pH = 5, T = 25 °C, UI = 20 kHz	90% 1 h	[13]
RhB (10 mg/L)	Ag_3_PO_4_–Fe_3_O_4_@AB (10 nm)	Cat = 0.1 g/L, H_2_O_2_ = 1.5 mM pH = 3.5, T = 25 °C, UI = 40–970 kHz	99.3% 1 h	[35]
AO 7 (35 mg/L)	GO–Fe_3_O_4_ NPs Fe_3_O_4_ NPs (10 nm)	Cat = 0.2 g/L, H_2_O_2_ = 22 mM pH = 3, T = 25 °C	98%, 3 h ~80%	[11]
MB (120−300 mg/L)	α-Fe_2_O_3_/SiO_2_	Cat = 1 g/L, H_2_O_2_ = 2 M pH = 3, T = 25 °C	99% 60 h	[23]
MB (50 mg/L) RhB (25 mg/L))	α-Fe_2_O_3_@Ti-tmSiO_2_	Cat = 0.25 g/L, H_2_O_2_ = 53 mM pH = 4, T = 50 °C	100%, 5.5 h 100%, 9h	[36]
AR 1 (200 mg/L)	Fe_3_O_4_ NPs (3.2 nm)	Cat = 4 g/L, H_2_O_2_ = 2 mM pH = 3.5, T = 25 °C	97.4% 1 h	This work

## Data Availability

Not applicable.

## References

[1] Singh K., Arora S. (2011). Removal of synthetic textile dyes from wastewaters: A critical review on present treatment technologies. Crit. Rev. Environ. Sci. Technol..

[2] Shaul G.M., Holdsworth T.J., Dempsey C.R., Dostal K.A. (1991). Fate of water-soluble azo dyes in the activated sludge process. Chemosphere.

[3] UNESCO (2012). The United Nations World Water Development Report 4: Managing Water Report under Uncertainty and Risk.

[4] Brillas E., Martínez-Huitle C.A. (2015). Decontamination of wastewaters containing synthetic organic dyes by electrochemical methods. An up dated review. Appl. Catal. B Environ..

[5] Anjaneyulu Y., Sreedhara Chary N., Suman Raj D.S. (2005). Decolorization of industrial effluents–available methods and emerging technologies—A review. Rev. Environ. Sci. Biotechnol..

[6] Florenza X., Sales Solano A.M., Centellas F., Martínez-Huitle C.A., Brillasa E., Garcia-Segura S. (2014). Degradation of the azo dye Acid Red 1 by anodic oxidation and indirectelectrochemical processes based on Fenton’s reaction chemistry. Relationship between decolorization, mineralization and products. Electrochim. Acta.

[7] Do Vale-Júnior E., Da Silva D.R., Fajardo A.S., Martínez-Huitle C.A. (2018). Treatment of an azo dye effluent by peroxi-coagulation and its comparison to traditional electrochemical advanced processes. Chemosphere.

[8] Javaid R., Qazi U.Y. (2019). Catalytic Oxidation Process for the Degradation of Synthetic Dyes: An Overview. Int. J. Environ. Res. Public Health.

[9] Hodges B.C., Cates E.L., Kim J.-H. (2018). Challenges and prospects of advanced oxidation water treatment processes using catalytic nanomaterials. Nat. Nanotechn..

[10] Munoz M., de Pedro Z.M., Casas J.A., Rodriguez J.J. (2015). Preparation of magnetite-based catalysts and their application in heterogeneous Fenton oxidation–A review. Appl. Catalys. B Environ..

[11] Zubir N.A., Yacou C., Motuzas J., Zhang X., Diniz da Costa J.C. (2014). Structural and functional investigation of graphene oxide–Fe3O4 nanocomposites for the heterogeneous Fenton-like reaction. Sci. Rep..

[12] Maharjan A., Dikshit P.K., Gupta A., Kim B.S. (2020). Catalytic activity of magnetic iron oxide nanoparticles for hydrogen peroxide decomposition: Optimization and characterization. J. Chem. Technol. Biotechnol..

[13] Wang N., Zhu L., Wang M., Wang D., Tang H. (2010). Sono-enhanced degradation of dye pollutants with the use of H2O2 activated by Fe3O4 magnetic nanoparticles as peroxidase mimetic. Ultrason. Sonochem..

[14] Robles H. (2014). Tannic Acid. Encyclopedia of Toxicology.

[15] Du X., Xu Y., Qin L., Lu X., Liu Q., Bai Y. (2014). Simple and Rapid Spectrophotometric Determination of Titanium on Etched Aluminum Foils. Am. J. Anal. Chem..

[16] Deng J., Wen X., Wang Q. (2012). Solvothermal in situ synthesis of Fe3O4-multiwalled carbon nanotubes with enhanced heterogeneous Fenton-like activity. Mater. Res. Bull..

[17] Cheera P., Karlapudi S., Sellola G., Ponneri V. (2016). A facile green synthesis of spherical Fe3O4 magnetic nanoparticles and their effect on degradation of methylene blue in aqueous solution. J. Mol. Liq..

[18] Bhattacharjee S., Mazumder N., Mondal S., Panigrahi K., Banerjee A., Das D., Sarkar S., Roy D., Kumar Chattopadhyay K. (2020). Size-modulation of functionalized Fe3O4: Nanoscopic customization to devise resolute piezoelectric nanocomposites. Dalton Trans..

[19] Ali S., Khan S.A., Yamani Z.H., Qamar M.T., Morsy M.A., Sarfraz S. (2019). Shape and size-controlled superparamagnetic iron oxidenanoparticles using various reducing agents and their relaxometric properties by Xigo acorn area. Appl. Nanosci..

[20] Zhang X., Niu Y., Meng X., Li Y., Zhao J. (2013). Structural evolution and characteristics of the phase transformations between α-Fe2O3, Fe3O4 and γ-Fe2O3 nanoparticles under reducing and oxidizing atmospheres. Cryst. Eng. Comm..

[21] Bibi I., Nazar N., Ata S., Sultan M., Ali A., Abbas A., Jilani K., Kamal S., Sarim F.M., Khan M.I. (2019). Green synthesis of iron oxide nanoparticles using pomegranate seeds extract and photocatalytic activity evaluation for the degradation of textile dye. J. Mater. Res. Technol..

[22] Razack S.A., Suresh A., Sriram S., Ramakrishnan G., ·Sadanandham S., Veerasamy M., Nagalamadaka R.B., ·Sahadevan R. (2020). Green synthesis of iron oxide nanoparticles using Hibiscus rosa-sinensis for fortifying wheat biscuits. SN Appl. Sci..

[23] Wu Z., Zhu W., Zhang M., Lin Y., Xu N., Chen F., Wang D., Chen Z. (2018). Adsorption and Synergetic Fenton-like Degradation of Methylene Blue by a Novel Mesoporous α-Fe2O3/SiO2 at Neutral pH. Ind. Eng. Chem. Res..

[24] Stoia M., Istratie R., Păcurariu C. (2016). Investigation of magnetite nanoparticles stability in air by thermal analysis and FTIR spectroscopy. J. Therm. Anal. Calorim..

[25] Chamritski I., Burns G. (2005). Infrared- and Raman-Active Phonons of Magnetite, Maghemite, and Hematite: A Computer Simulation and Spectroscopic Study. J. Phys. Chem. B.

[26] De Faria D.L.A., Venaüncio Silva S., De Oliveira M.T. (1997). Raman Microspectroscopy of Some Iron Oxides and Oxyhydroxides. J. Raman Spectrosc..

[27] Shebanova O.N., Lazor P. (2003). Raman spectroscopic study of magnetite (FeFe2O4): A new assignment for the vibrational spectrum. J. Sol. Stat. Chem..

[28] Guin D., Manorama S.V. (2008). Room temperature synthesis of monodispersed iron oxide nanoparticles. Mater. Lett..

[29] Herrera-Becerra R., Rius J.L., Zorrilla C. (2010). Tannin biosynthesis of iron oxide nanoparticles. Appl. Phy. A.

[30] Morgan B., Lahav O. (2007). The effect of pH on the kinetics of spontaneous Fe(II) oxidation by O2 in aqueous solution–basic principles and a simple heuristic description. Chemosphere.

[31] Pignatello J.J., Oliveros E., MacKay A. (2006). Advanced Oxidation Processes for Organic Contaminant Destruction Based on the Fenton Reaction and Related Chemistry. Crit. Rev. Environ. Sci. Technol..

[32] Shoniya T., Sreekanth R., Sijumon V.A., Usha K.A., Aravinda K.C.T. (2014). Oxidative degradation of Acid Red 1 in aqueous medium. Chem. Eng. J..

[33] Gao L., Zhuang J., Nie L., Zhang J., Zhang Y., Gu N., Wang T., Feng J., Yang D., Perrett S. (2007). Intrinsic peroxidase-like activity of ferromagnetic nanoparticles. Nat. Nanotechnol..

[34] Tong J., Xu Z., Bian Y., Niu Y., Zhang Y., Wang Z. (2019). Flexible and smart fibers decorated with Ag nanoflowers for highly active surface-enhanced Raman scattering detection. J. Raman Spectrosc..

[35] Jun B.-M., Kim Y., Yoon Y., Yea Y., Park C.M. (2020). Enhanced sonocatalytic degradation of recalcitrant organic contaminants using a magnetically recoverable Ag/Fe-loaded activated biochar composite. Ceram. Intern..

[36] Lv Q., Li G., Sun H., Kong L., Lu H., Gao X. (2014). Preparation of magnetic core/shell structured c-Fe2O3@Ti-tmSiO2 and its application for the adsorption and degradation of dyes. Microporous Mesoporous Mater..

